# Mechatronic Design and Development of a Lower-Limb Exoskeleton System Based on Knee Joint Biomechanical Principles Using Electro-Pneumatic Actuation with an Embedded EMG Controller for Experimental Validation in Elderly Gait Rehabilitation Support

**DOI:** 10.3390/bioengineering13060644

**Published:** 2026-05-29

**Authors:** Adrian Nacarino, Bryan Sanchez, Sandra Charapaqui, Renzo Charapaqui, Renzo R. Maldonado-Gómez, Leslie M. Mendoza-Arias, Daira de la Barra, Cristina Ccellcaro, Ricardo Palomares, Jose Cornejo, Mariela Vargas, Robert Castro, Jorge Cornejo

**Affiliations:** 1 Instituto de Investigaciones en Ciencias Biomédicas, Universidad Ricardo Palma, Lima 15039, Peru; 202111392@urp.edu.pe (B.S.); 202020374@urp.edu.pe (S.C.); 201512659@urp.edu.pe (R.C.); 202120753@urp.edu.pe (R.R.M.-G.); 202120263@urp.edu.pe (L.M.M.-A.); 202120213@urp.edu.pe (D.d.l.B.); 202120547@urp.edu.pe (C.C.); jose.cornejoag@urp.edu.pe (J.C.); mariela.vargas@urp.edu.pe (M.V.); 2 Research Group of Advanced Robotics and Mechatronics (GI-ROMA), Ricardo Palma University, Lima 15039, Peru; ricardo.palomares@urp.edu.pe; 3 Professional School of Mechatronics Engineering, Ricardo Palma University, Lima 15039, Peru; robert.castro@urp.edu.pe; 4 Space Generation Advisory Council, 1010 Vienna, Austria; 5 Biomimetic Engineering and Aerospace Mechatronics (BEAM) Laboratory, Betta International Corporation, Lima 15026, Peru; 6 Center for Space Systems (C-SET), Betta Aerospace, Betta International Corporation, Lima 15026, Peru; 7 Mayo Clinic, Jacksonville, FL 32224, USA; jcornejo_17@ieee.org

**Keywords:** knee exoskeleton, electromyography, electro-pneumatic, lower-limb orthosis, gait assistance, elderly rehabilitation, embedded control systems, 3D printing

## Abstract

Stroke is the second leading cause of death globally and a major contributor to lower-limb disability, affecting gait, balance, and functional independence in elderly populations. While robot-assisted rehabilitation has demonstrated effectiveness in motor recovery, access remains limited due to high costs and geographic barriers, particularly in Latin America. This study presents ExoKnee, a low-cost knee exoskeleton designed through biomimetic principles and 3D-printed fabrication as a proof-of-concept device targeting gait rehabilitation in elderly adults. The system integrates a single-degree-of-freedom pneumatic actuator controlled by electromyography (EMG) signals from the quadriceps muscle, enabling knee flexion and extension (90° to 180°). The design was evaluated through finite element analysis and dynamic simulations in MATLAB/Simulink R2024a under constant, stepwise, and sinusoidal reference inputs in a digital-twin environment. Expert validation using the Content Validity Coefficient yielded a mean score of 0.8747, reflecting preliminary expert agreement on the conceptual design’s coherence and relevance. The prototype demonstrated controlled movements through a 6-bar pneumatic system with EMG-triggered relay activation, validated at the proof-of-concept level through simulation and single-subject threshold calibration. ExoKnee addresses critical gaps by offering an anthropometrically informed, biosignal-driven, and locally manufacturable rehabilitation platform for low- and middle-income countries, pending clinical validation. Future work will focus on clinical trials and adaptive EMG control strategies.

## 1. Introduction

Globally, stroke is the second leading cause of death and the third leading cause of disability. Following a stroke, complications affecting the lower limbs are highly prevalent: lower-limb spasticity affects approximately 25% of patients, increasing to up to 40% in those with paresis, thereby directly compromising gait and functional mobility [1]. Concomitantly, more than 50% of stroke survivors present balance impairments, leading to postural instability, an increased risk of falls, and persistent limitations in mobility. Clinical studies have shown that lower-limb motor recovery after stroke depends on both the integrity of the corticospinal tract and the quality and composition of leg muscles, including knee flexion and extension strength, which directly influence gait speed, stability, and functional independence [2]. In this context, robot-assisted rehabilitation has emerged as an effective strategy for lower-limb functional recovery after stroke by enabling intensive and repetitive gait-oriented movements. Technical and clinical reviews indicate that robotic lower-limb rehabilitation systems can improve motor function, walking ability, and balance, promoting gait pattern recovery and reducing functional disability in post-stroke patients [3]. The evidence suggests that this complementary approach may overcome some limitations of conventional therapy by providing more standardized and personalized training [4].

Compared with other regions of the world, Latin America and the Caribbean show a pooled stroke incidence of 255 cases per 100,000 person-years, with higher rates in men than in women, particularly among vulnerable populations with limited access to high-quality rehabilitation services. This epidemiological burden translates into a high proportion of stroke survivors with persistent motor disability, especially affecting the lower limbs, where gait and balance impairments represent major determinants of post-stroke functional dependence; this can be seen in the fact that approximately 44% of patients present motor weakness in the lower limbs and that between 46% and 58% are unable to walk independently after the event [5,6]. Despite this high burden, post-stroke rehabilitation coverage remains limited, and many patients face economic, geographic, and logistical barriers that restrict access to continuous and effective therapy. Robotic rehabilitation may help mitigate disparities in healthcare access; however, its high cost limits the number of patients who can benefit from it. In response, the development of low-cost devices through 3D printing offers a viable solution, enabling the replacement of expensive metal alloys while maintaining functionality and ergonomics. This approach aligns with recent advances in anatomical engineering and additive manufacturing, which have demonstrated great potential for developing customized, patient-specific medical devices that enhance clinical accessibility and performance [7].

In Peru, stroke is the second leading cause of death and one of the main causes of premature mortality and disability, particularly among older adults and men, making it a high-cost disease. In rural areas, a stroke prevalence of 7.05 per thousand inhabitants has been reported, highlighting the substantial burden of this condition in communities with limited access and the significant economic and geographic barriers that hinder effective treatment. Rehabilitation programs are concentrated in Lima and a few regional capitals, leaving significant gaps between urban and rural areas. Moreover, there is a shortage of functional assistive devices such as prostheses and exoskeletons. Consequently, the local development of low-cost, patient-adaptable 3D-printed lower-limb exoskeletons emerges as a priority need to expand access to rehabilitation, complement conventional therapeutic approaches, and improve the quality of life of stroke survivors [8].

Beyond rehabilitation, recent studies in biomedical soft robotics have explored applications in extreme environments such as space, where muscle atrophy and circulatory impairment are critical challenges [9]. For instance, soft robotic systems have been proposed to provide mechanical stimulation and monitor muscle performance in microgravity, achieving up to 89% energy optimization and a safety factor of 2.75 in computational simulations [10]. These innovations illustrate how bioinspired and energetically efficient robotic systems can support both physiological recovery and human performance in a complex environment [11]. Following this trend, the present study introduces ExoKnee, a proof-of-concept pneumatic knee exoskeleton designed using biomimetic and anthropometric principles, targeting future gait assistance and mobility improvement in elderly adults through neuromuscular control and 3D-printed fabrication.

Regarding mechanical design, consistent trends were identified across actuation technologies, transmission mechanisms, structural materials, and design methodologies. Most lower-limb exoskeletons rely on brushless DC motors and servo actuators due to their high torque-to-weight ratio and controllability [12,13,14,15], although alternative solutions, including stepper motors, SMA-based actuation, and variable-stiffness mechanisms, have also been explored [16,17,18]. Transmission systems frequently employ harmonic drives, ball-screw mechanisms, and tendon-driven or Bowden-cable architectures, which remain dominant due to their low inertia and ability to replicate natural joint kinematics [12,19,20,21]. Structurally, lightweight polymers fabricated through additive manufacturing are widely used in wearable components, whereas aluminum alloys and composite materials such as Carbon Fiber-Reinforced Polymer (CFRP) or Kevlar are preferred in high-load regions to improve stiffness and durability [15,22,23,24]. Reported device masses range from ultralight configurations below 1.5 kg to rigid exoskeletons exceeding 10 kg, reflecting the trade-off between portability and load assistance [18,25,26]. Design and simulation are commonly performed using computed aided design (CAD); multibody modeling platforms such as SolidWorks 2023, Autodesk Inventor Professional 2024, and MATLAB R2024a; and dynamic simulation tools, highlighting the integration of biomechanical modeling and mechanical optimization in modern exoskeleton development [12,14,27].

Regarding electronic architecture, most exoskeleton systems integrate multimodal sensing combining inertial, kinematic, force, and electromyographic measurements to enable real-time monitoring and adaptive control. Common sensing solutions include IMUs, incremental encoders, force and pressure sensors, torque transducers, and wireless EMG modules [12,16,24,26,28]. Embedded control is typically implemented using microcontroller-based platforms such as Arduino, STM32, or ESP32 boards, while more computationally demanding applications incorporate DSP units, real-time controllers, or embedded AI platforms [17,21,22]. Motor control is generally achieved through dedicated drivers and power electronics modules, supported by Li-ion battery systems with integrated safety circuitry [26,29]. Electronic subsystems consistently prioritize real-time signal acquisition, reliable actuation control, and modular hardware integration suitable for rehabilitation robotics.

Regarding informatics and software frameworks, most rehabilitation exoskeletons employ MATLAB-based environments for modeling, simulation, signal processing, and controller design, frequently complemented by LabVIEW for real-time data acquisition and system monitoring [13,25,28]. Embedded control is commonly implemented using C/C++ within Arduino-based or microcontroller-specific development environments [16,17,30]. More advanced robotic applications incorporate ROS-based frameworks for distributed communication and modular control, while biomechanical analysis is often supported by simulation platforms such as OpenSim and motion capture integration tools [14,21,26]. Overall, the literature shows a strong preference for hybrid software ecosystems combining high-level modeling platforms with low-level embedded programming to ensure real-time responsiveness and biomechanical accuracy.

The literature on lower-limb exoskeletons shows clear benefits in reducing muscular effort, improving gait metrics, and assisting sit-to-stand transitions, but most studies remain preliminary: they rely on small samples, single-session tests, healthy young participants, limited degrees of freedom, and treadmill-based assessments and lack long-term validation of comfort, durability, and autonomy. Few devices are truly adapted to the anthropometry and needs of older adults, especially in low-resource contexts, and many prototypes offer insufficient torque, incomplete biomechanical alignment, or no real-time adaptive control in realistic environments.

Recent studies have specifically addressed knee exoskeletons designed for real-world assistance and rehabilitation. Devine TM et al. (2025) investigated the clinical feasibility of a robotic knee exoskeleton incorporating dual control strategies to assist gait in individuals with neuromuscular impairments, highlighting its potential for overground application beyond treadmill-based environments [31]. Similarly, Jin S et al. (2025) developed a bionic knee exoskeleton using rope-driven artificial muscles capable of dynamically modulating joint stiffness, demonstrating improved comfort and reduced quadriceps activation as measured by electromyography [32]. Lin Wu et al. (2024) presented a knee exoskeleton featuring a compliant actuator and variable impedance control for post-stroke rehabilitation, achieving smooth human–robot interaction and precise assistance [33]. In addition, Wang et al. (2023) analyzed knee flexion assistance strategies that incorporate energy storage and release mechanisms throughout the gait cycle, emphasizing the importance of phase-specific support to optimize biomechanical efficiency [34]. Furthermore, Harsh-Harikrishnan S et al. (2025), in a recent review of knee and ankle exoskeleton systems, highlighted the ongoing transition toward lightweight, semi-rigid, and soft designs that prioritize portability, adaptability, and ergonomic human–robot interaction, while also noting persistent challenges related to durability, real-time adaptive control, and long-term clinical validation [35].

In recent years, the development of knee exoskeletons has attracted growing attention due to their potential to improve gait performance and facilitate rehabilitation. Bryan et al. (2021) evaluated a multi-joint hip–knee–ankle exoskeleton in which knee assistance was adjusted according to walking speed, showing that modulating knee torque in relation to gait velocity can significantly reduce metabolic cost while enhancing overall walking efficiency [36]. Using a different strategy, Etenzi et al. (2020) investigated a passive-elastic knee–ankle device that stores mechanical energy during specific phases of the gait cycle and releases it when required, reducing the energetic demand of walking without the need for active actuation and highlighting the advantages of lightweight mechanical solutions [37]. In a more adaptive framework, de Miguel Fernandez et al. (2023) analyzed a unilateral active knee exoskeleton capable of delivering both assistive and resistive torques, reporting improvements in knee flexion amplitude and neuromuscular activation patterns that support functional recovery and motor retraining [38]. From a mechanical design perspective, Laubscher et al. (2021) emphasized the importance of anthropometric parameterization to achieve proper alignment between the exoskeleton and the biological knee joint, thereby improving torque transmission efficiency and user comfort [39]. Additionally, Ma et al. (2021) proposed a hip–knee coupling mechanism based on offset theory that enables coordinated energy transfer between joints and enhances phase-specific assistance, contributing to smoother joint interaction and reduced mechanical discontinuities during walking [40]. The main characteristics, advantages and disadvantages of the reviewed knee exoskeletons are presented in Table 1.

These gaps highlight the need for accessible, well-fitted, and clinically validated knee exoskeletons designed for elderly users. ExoKnee directly addresses this need through an anthropometrically informed and modular mechanical design, EMG-based intent detection, and a pneumatic actuation system optimized for knee flexion–extension. The system integrates MATLAB/Simulink dynamic modeling and simulation for design validation, leading to a functional 3D-printed prototype evaluated in a digital-twin environment and subjected to single-subject EMG threshold calibration as an initial feasibility test. It is important to note that no formal human-subject gait trials have been conducted at this stage; clinical validation with elderly users remains as future work. This approach addresses the main deficiencies identified in the literature: lack of elderly-specific adaptation, limited use of biosignals for voluntary control, and insufficient low-cost, locally manufacturable solutions for real-world rehabilitation.

The manuscript is organized as follows: Section 2 describes materials and methods for the bio-mechatronics design of the knee exoskeleton for gait support, including simulation of the control system. Section 3 focuses on the system manufacturing and integration, while Section 4 presents the expert validation results through Content Validity Coefficient analysis, evaluating system functionality, usability, and therapeutic potential. In Section 5, the manuscript ends with conclusions and further work.

## 2. Materials and Methods

**Figure 1:**
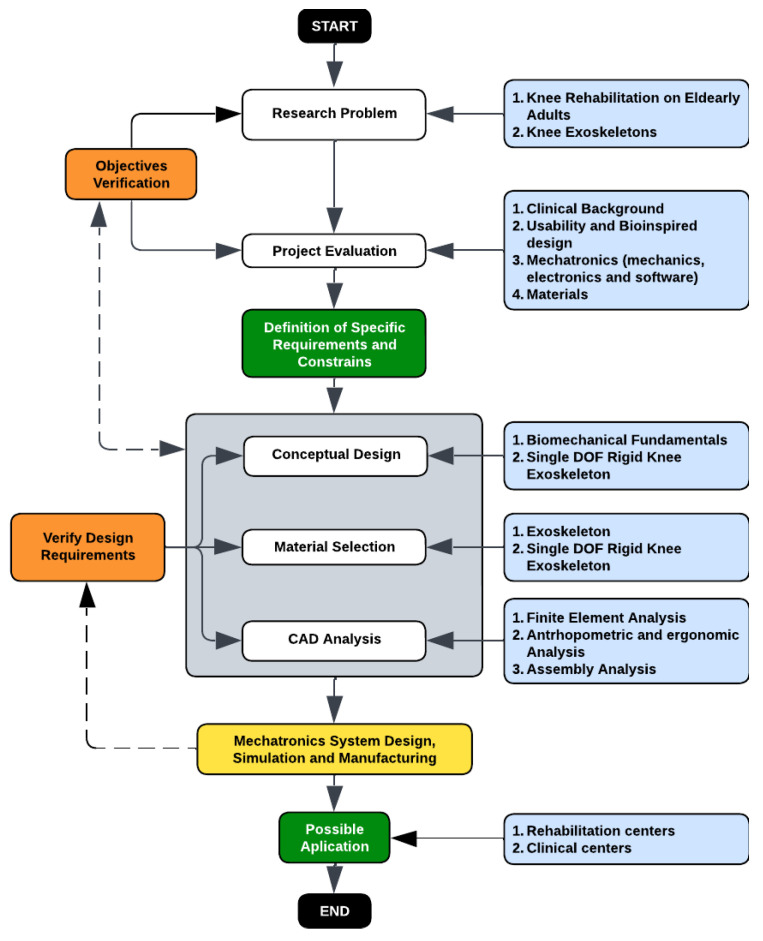
Bio-design Methodology of ExoKnee.

The research began by identifying the core problem associated with knee rehabilitation in elderly adults and reviewing the state of the art in knee exoskeletons, establishing the clinical and technological motivations for the project. Following this, the project evaluation stage integrated clinical background, usability considerations, and bioinspired design principles, together with an assessment of the mechanical, electronic, and software components relevant to mechatronic development, as well as the selection of suitable materials. This evaluation enabled the verification of the initial objectives and informed the subsequent definition of specific design requirements and constraints. Based on these criteria, the conceptual design was developed through an analysis of lower-limb biomechanics and the selection of a single-degree-of-freedom rigid knee exoskeleton architecture. Material selection was carried out for the structural components of the exoskeleton, ensuring compatibility with the intended mechanical performance and user comfort. This was followed by the CAD analysis phase, which included finite element simulations, anthropometric and ergonomic assessment of elderly users, and assembly verification. The final stage encompassed the mechatronic system design, simulation, and manufacturing of the prototype, establishing the foundation for future application in rehabilitation and clinical centers (Figure 1).

### 2.1. Mechatronics Design and Simulation

This research developed a single-degree-of-freedom knee exoskeleton designed to support rehabilitation in elderly adults. The system uses a rigid structure adapted to lower-limb anthropometry and an electro-pneumatic actuator that generates controlled knee flexion and extension. User intent and joint motion are monitored through an EMG sensor, which provides real-time inputs to the embedded control system. The overall mechatronic design combines the structural mechanism, sensing, actuation and control into a proof-of-concept prototype conceived as a foundation for future application in rehabilitation and clinical settings.

#### 2.1.1. Mechanics

**Figure 2:**
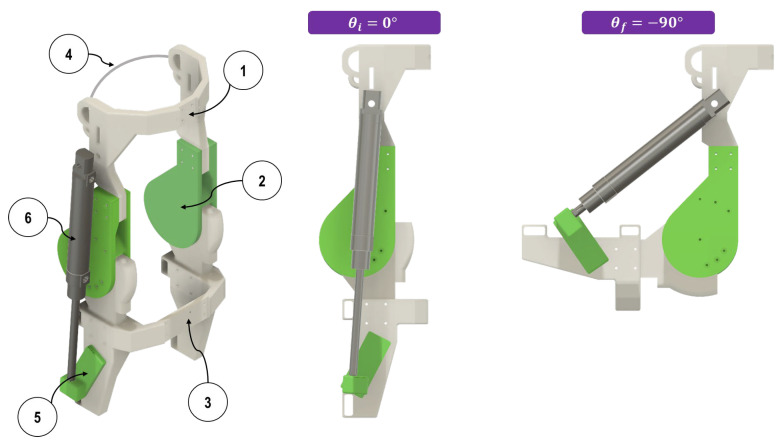
ExoKnee parts and range of motion. Quadriceps support (1), rotational knee joint (2), calf support (3), (4) hook-and-loop fastener, configurable linkage (5), and pneumatic cylinder (6).

Figure 2 illustrates the structural components and actuation configuration of the proposed knee exoskeleton. The quadriceps support (1) provides proximal fixation to stabilize the thigh segment, while the rotational knee joint (2) enables controlled flexion and extension around a single anatomical axis. The calf support (3) secures the distal segment of the leg and ensures proper alignment during movement. The configurable linkage (5) connects the pneumatic actuator to the lower segment and allows the linear motion of the rod to translate into the rotational motion required for knee flexion and extension. The pneumatic cylinder (6) serves as the primary actuator, generating the force needed to assist knee movement. The two right-side views show the actuator configuration at the initial angle *θ_i_* = 180° and at the final angle *θ_f_* = 90°, demonstrating the full operational range of the mechanism.

**Figure 3:**
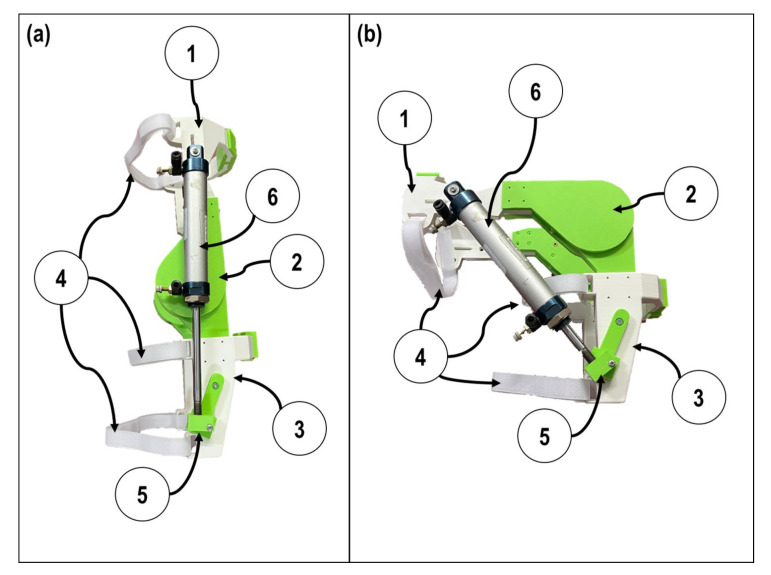
Mechanical prototyping of ExoKnee using PLA+ material with 0.27 mm layer thickness and 78% infill density via Creality Print 6.1. Quadriceps support (1), rotational knee joint (2), calf support (3), hook-and-loop fasteners (4), configurable linkage (5), and FESTO DSNU-S-12-100-P-A-MQ pneumatic actuator (6) with 100 mm stroke. The single-degree-of-freedom design driven by the pneumatic piston enables controlled sagittal plane motion (90° to 180°), simplifying control architecture and reducing mechanical complexity while maintaining biomechanically relevant knee flexion–extension for elderly gait rehabilitation. (**a**) Extension position; (**b**) flexion position.

For the next phase, the physical integration of the system was made. Initially, the quadriceps support (1), the rotational knee joint (2), calf support (3), and the configurable linkage (5) were exported from Fusion 360 to Creality Print 6.1 for 3D printing. Based on previous studies [41], printing parameters were set to achieve maximum tensile strength (33.7 MPa): layer thickness of 0.27 mm and infill of 78%. Tree supports for corners less than 20° were considered, as well as base adhesion to the heated bed, and favorable results were achieved. Additionally, the hook-and-loop fasteners (4) were considered for proper adhesion with the lower limb. The pneumatic actuator (6) was a FESTO DSNU-S-12-100-P-A-MQ with a linear displacement of 100 mm (Figure 3).

The fabricated prototype has a total structural mass of approximately 0.18 kg for the 3D-printed components, with the FESTO pneumatic actuator adding approximately 0.1 kg, resulting in a lightweight wearable assembly. However, the current configuration requires an external compressed air supply at 6 bars, which limits immediate portability. Miniaturized pneumatic supply solutions will be evaluated in future iterations to enable community-level use. Regarding safety, beyond the structural margins demonstrated through FEA, future designs should incorporate fail-safe valve configurations, emergency stop mechanisms, and software-based limits to prevent unintended activation or excessive extension. It should be acknowledged that simplifying the knee joint to a single-axis hinge represents a biomechanical limitation of this proof-of-concept design. The human knee exhibits a moving instantaneous center of rotation throughout the flexion–extension arc, which a fixed-axis mechanism cannot fully replicate. This simplification may introduce misalignment forces at the interface, particularly relevant for elderly or post-stroke users who are sensitive to joint loading and discomfort. Addressing this limitation through polycentric or cam-based mechanisms that track the instantaneous center of rotation is recognized as an important direction for future development.

#### 2.1.2. Geometric, Kinematic and Dynamic Model

**Figure 4:**
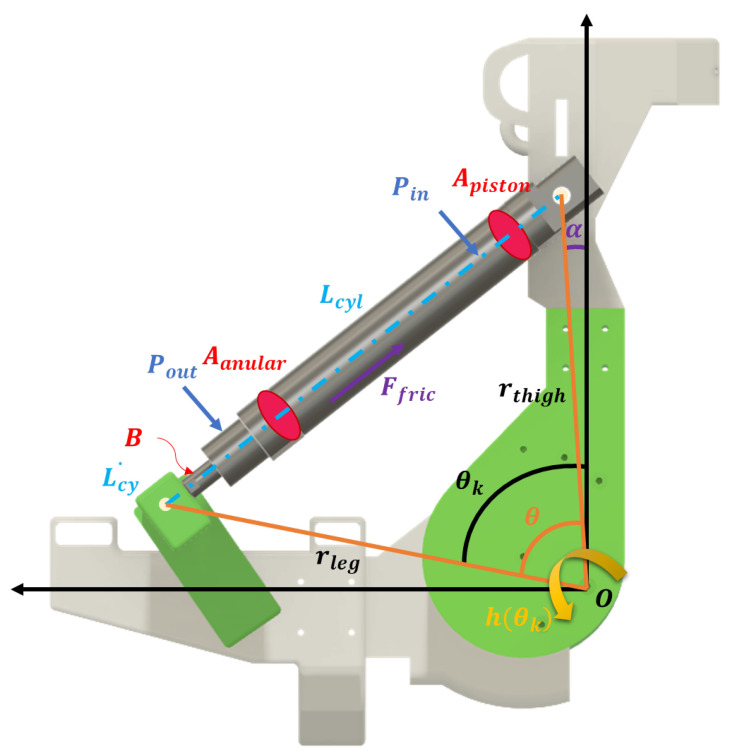
Mechanical modeling of ExoKnee with associated physical parameters for a single-degree-of-freedom, electro-pneumatic actuator.

To validate the design, an analytical model was developed to describe the dynamics of the bio-mechatronic system [42,43]. The exoskeleton is modeled as a single degree-of-freedom (1-DoF) mechanism pneumatically actuated and kinematically coupled to the user’s lower limb (Figure 4). Force transmission is achieved through a linear pneumatic cylinder articulated between the quadriceps and the calf support. The geometric relationship between the actuator length $L_{cyl}$ and the knee angle $\theta_{k}$ (where $\theta_{k}=180{}^{\circ}$ corresponds to full extension) is determined through vector analysis of the mechanical triangle formed by the knee rotation center O and the anchoring points A and B (Equation (1)).

(1) $$L_{cyl}(\theta_{k})=\sqrt{{r_{thigh}}^{2}+{r_{leg}}^{2}-2r_{thigh}r_{leg}\cos\left(\theta \right)}$$

where $r_{thigh}$ and $r_{leg}$ are the link lengths from the rotation axis to the anchoring points, and $\theta =\theta_{k}-\alpha$, and *α* is the structural offset angle. The configurable linkage design allows for mechanical adjustment of these anchoring lengths to accommodate different user dimensions, providing a basis for modular anthropometric adaptation. The structural offset angle *α* can similarly be adjusted during assembly to align with individual knee geometry. A formal sizing protocol covering the full range of elderly adult dimensions defined in ISO 7250-1:2017 [44], including alignment tolerance analysis and interface pressure evaluation, is planned as part of future clinical development. The actuator extension velocity $\dot{L_{cyl}}$ is related to the joint angular velocity $\dot{\theta_{k}}$ through the geometric Jacobian $J(\theta_{k})$, as shown in Equation (2).

(2) $$\dot{L_{cyl}}=J\left(\theta_{k}\right)\dot{\theta_{k}}=\left(\frac{r_{thigh}r_{leg}\sin\left(\theta_{k}-\alpha \right)}{L_{cyl}(\theta_{k})}\right)\dot{\theta_{k}}$$

The dynamics of the combined system during the swing phase of gait are modeled using the Euler–Lagrange formulation, considering inertial, gravitational, and frictional effects, as shown in Equation (3).

(3) $$\tau_{act}=I_{eq}\left(\theta_{k}\right)\ddot{\theta_{k}}+B\dot{\theta_{k}}+\tau_{grav}\left(\theta_{k}\right)+\tau_{ext}$$

where $\tau_{act}$ is the effective torque applied by the exoskeleton. The gravitational term $\tau_{grav}$ is defined as Equation (4).

(4) $$\tau_{grav}\left(\theta_{k}\right)=M_{sys}gd_{cm}\sin\left(\theta_{k}\right)$$

Here, $M_{sys}$ represents the combined mass of the lower segment (user’s leg + exoskeleton structure), and $d_{cm}$ the distance to the center of mass. The combined center of mass was calculated using the ExoKnee’s mass distribution from CAD modeling and anthropometric data following ISO 7250-1:2017 for the leg segment. The current design targets average adult anthropometry as defined by ISO 7250-1:2017. Formal sizing protocols covering a range of user dimensions, alignment tolerance analysis, and interface pressure evaluation are recognized as necessary steps for clinical deployment and are planned as part of future development. The assistance torque $\tau_{act}$ generated by the pneumatic cylinder depends on the differential pressure and the instantaneous geometry, as shown in Equation (5).

(5) $$\tau_{act}=F_{pneu}h(\theta_{k})$$

where $h(\theta_{k})$ is the effective moment arm derived from the Jacobian. The pneumatic force $F_{pneu}$ is modeled as Equation (6).

(6) $$F_{pneu}=\left(P_{in}A_{piston}-P_{out}A_{anular}\right)-F_{fric}\mathrm{sgn}\left(\dot{L_{cyl}}\right)$$

The physical and geometric parameters used for the simulation and validation of the model are detailed in Table 2. These values correspond to the dimensions of the fabricated prototype and to standard anthropometric data for an average adult, corresponding to half the normative segment lengths for the thigh and shank, respectively, in an average adult. The structural offset angle α was determined experimentally during the mechanical design phase, measured directly from the fabricated prototype after assembly. The combined segment mass $M_{sys}$ represents an initial approximation of the lower leg and exoskeleton structure mass; a refined value based on Plagenhoef et al. [42] anthropometric data will be incorporated in future simulation iterations. The distance to the center of mass $d_{cm}$ was estimated from an initial CAD-based mass distribution analysis of the prototype. The equivalent inertia $I_{eq}$ was estimated using a first-order rigid segment model of the form $I=ML^{2}$, where M is the segment mass and L is the distance from the joint axis to the center of mass, following standard biomechanical approximations; formal experimental identification is deferred to future work. The viscous friction coefficient B was treated as a variable parameter rather than a fixed value, consistent with FESTO’s design philosophy of minimizing friction in their pneumatic actuators. As no specific coefficient is provided in the DSNU-S series datasheets, B was incorporated as a tunable parameter to assess simulation sensitivity, with experimental characterization deferred to future work. The piston diameter $D_{p}$ actuator stroke S, and nominal operating pressure $P_{nom}$ were obtained directly from the FESTO DSNU-S-12-100-P-A-MQ datasheet.

#### 2.1.3. Finite Element Analysis

**Figure 5:**
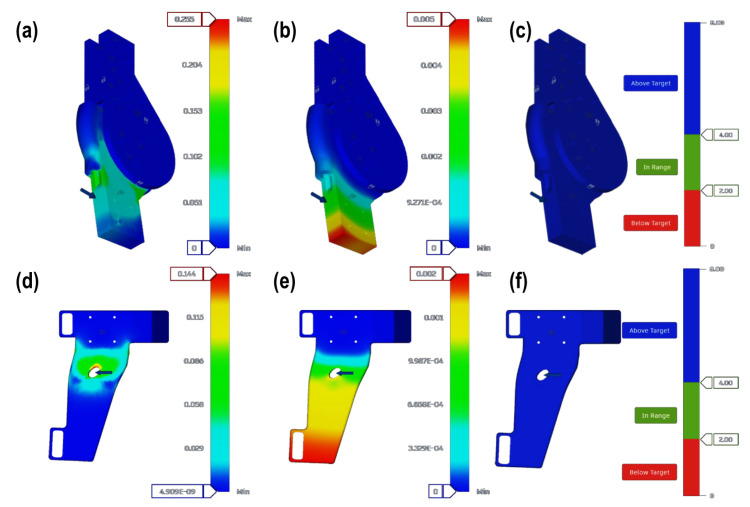
Finite element analysis results for ExoKnee components. (**a**) Von Mises stress distribution in knee joint under 25 N load, showing peak stress at bearing interface; (**b**) Displacement field of knee joint structure; (**c**) Safety factor distribution across knee mechanism; (**d**) Stress distribution in calf support under 50 N pneumatic force, with maximum values at central attachment region; (**e**) Displacement behavior of calf support assembly; (**f**) Safety factor evaluation confirming structural adequacy for operational cycles.

In the analysis, two groups were examined: one for the rotational knee joint and another for the calf support, which bears the weight of the knee. Static finite element analyses were conducted to determine von Mises stress, total displacements (x, y, z), and safety factors. For the first case, a force of 25 N (presented as a blue arrow) was applied to the lower part of the knee joint, representing the average load each joint must withstand [45], where the lower leg mass corresponds to approximately 4–5 kg (~40–50 N). The selected value represents a conservative partial loading condition transmitted through the joint structure. For the second group, a force of 50 N (presented as a blue arrow) was applied, corresponding to the conventional working pressure of the pneumatic actuator. These load cases were selected to reflect physiologically relevant operational conditions of the ExoKnee during assisted gait. Figure 5a shows that the maximum von Mises stress is 0.255 MPa, located at the bearing that enables rotational movement of the knee joint. Figure 5b illustrates that the maximum displacement is 0.005 mm at the lower section of the knee joint. Figure 5c demonstrates that the entire knee joint maintains a safety factor of 8, which is essential for biomedical devices. For the calf support assembly, Figure 5d reveals a maximum von Mises stress of 0.144 MPa at the central region, where the pneumatic actuator exerts the force to contract the knee, with a minimum value of 4.909 × 10^−9^ MPa. Figure 5e shows a maximum displacement of 0.002 mm observed at the lower portion of the calf support. Similarly, Figure 5f indicates a safety factor of 8, calculated based on the material’s yield strength. This value is satisfactory for the application as it accounts for dynamic loading conditions, manufacturing tolerances, and the critical safety requirements of a human-contact wearable device.

Regarding the finite element analysis boundary conditions, the rotational knee joint was fixed at its upper mounting interface, simulating attachment to the quadriceps support, while the 25 N load was applied at the lower articular surface. For the calf support, fixed constraints were applied at the strap attachment points, with the 50 N pneumatic force applied at the central actuator interface. For heavier patients or higher load scenarios, structural reinforcement through increased infill density beyond 78%, addition of internal ribbing, and replacement of PLA+ with PETG or carbon fiber-reinforced filaments have been identified as viable design adaptations. Additionally, the analysis assumed homogeneous isotropic material properties for PLA+, which represents a known simplification; fused deposition modeled parts exhibit anisotropic behavior due to layer orientation, interlayer adhesion variability, and sensitivity to humidity and cyclic loading. Consequently, the reported safety factors may overestimate actual structural margins under real operating conditions. Future analyses should incorporate orthotropic material models calibrated from tensile testing of printed specimens at relevant orientations, alongside dynamic loading scenarios and cyclic fatigue analysis, to provide more conservative and realistic structural predictions.

#### 2.1.4. Electronics Design and Control System

For the control architecture of the proposed knee exoskeleton, EMG electrodes were placed on the quadriceps to capture the muscle activation signal, which was conditioned by the AD8832 EMG module (1), powered by ±9 V batteries (2), and transmitted to an Arduino Uno (3). The EMG signal was acquired using the 10-bit ADC of the Arduino Uno, which provides a measurement range from 0 to 1023 ADC units. The microcontroller interpreted the processed signal as an ON-OFF command based on a predefined activation threshold. Prior to testing, a maximum voluntary contraction (MVC) trial was performed on one of the authors to calibrate the system. During this contraction, peak EMG values ranged approximately between 500 and 700 ADC units. The threshold was intentionally set at 500 ADC units to ensure reliable triggering under the adopted binary (ON-OFF) control strategy. Since this prototype was conceived as a proof-of-concept system, a fixed subject-specific calibration was considered sufficient to guarantee consistent activation.

**Figure 6:**
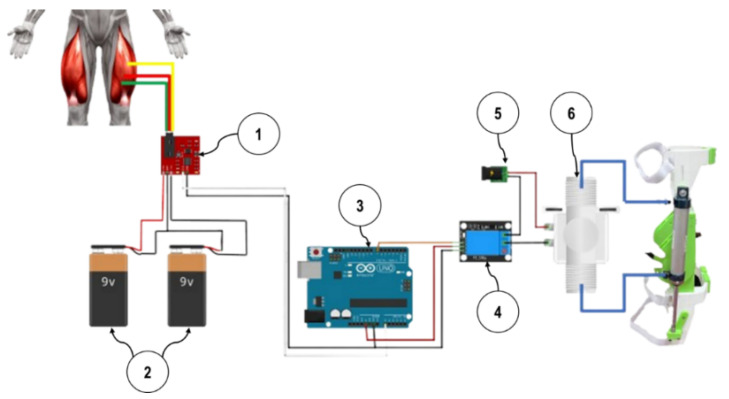
Circuit diagram of the electronic system. AD8832 (1), −9 V and 9 V batteries (2), Arduino Uno (3), relay module (4), 24 V power supply (5), solenoid valve (6). The blue lines indicate pneumatic air flow.

When the activation level exceeded this threshold, the Arduino (3) triggered the relay module (4) for a fixed maximum duration of 5 s. This duration corresponds to the electrical activation window of the system, not to the mechanical extension time of the pneumatic actuator. Once activated, the relay (4) allowed the 24 V power supply (5) to energize the solenoid valve (6). The electrical response of the relay-valve chain occurs within milliseconds, enabling compressed air to be delivered almost immediately to the pneumatic cylinder. The actual piston extension time depends on supply pressure, airflow rate, and external load conditions and is significantly shorter than the 5 s activation window. If no activation was detected, the relay module (4) remained open, the solenoid valve (6) stayed closed, and the cylinder remained unpressurized, keeping the knee in the flexed position (Figure 6). For future multi-subject or clinical applications, normalization strategies (e.g., relative to MVC percentage) and adaptive proportional control schemes will be implemented to enable automatic calibration and improved inter-subject scalability.

It is further acknowledged that EMG signal characteristics differ substantially between healthy individuals and the target population of elderly or post-stroke users, where factors such as muscle weakness, spasticity, co-contraction, increased skin impedance, and signal variability may significantly affect threshold-based detection reliability. The single-subject calibration performed here is therefore not generalizable to clinical populations and serves exclusively as a proof-of-concept demonstration of the control logic. Population-specific calibration protocols, including normalization relative to individual MVC percentage and adaptive thresholding strategies, will be essential prerequisites for any future clinical application.

Before describing the simulation framework, it is important to distinguish between the two control levels present in this work. At the hardware level, the fabricated prototype operates under a binary ON/OFF threshold-based strategy, where EMG signal detection triggers relay activation as described in the previous section. At the simulation level, a closed-loop trajectory-tracking architecture was developed in MATLAB R2024a using Simscape Multibody to characterize the dynamic behavior of the system under idealized continuous reference inputs. This simulation was not designed to replicate the binary hardware controller, but rather to evaluate the system’s dynamic response as a foundation for future closed-loop control implementation.

Regarding the hardware control strategy, the binary ON/OFF architecture has inherent limitations for gait assistance applications. This approach does not support phase-specific torque modulation, smooth force delivery proportional to muscular effort, or safe mid-cycle interruption—characteristics recognized as important requirements for naturalistic gait assistance in elderly users. The current implementation is therefore appropriate only as a proof-of-concept demonstration of EMG-triggered pneumatic activation and not as a clinically deployable gait assistance strategy. Future control architectures will incorporate proportional EMG-driven pressure modulation and gait-phase detection to address these limitations.

The closed-loop control architecture is implemented for the knee exoskeleton system, where the desired joint angle $\theta_{ref}$ is defined as the reference input. The reference signal, initially expressed in degrees, is converted to radians. The output corresponds to the pneumatic actuator displacement, which serves as the input to the dynamic model of the assisted knee joint. This model captures the nonlinear exoskeleton dynamics, including the variable moment arm derived from the geometric Jacobian, viscous damping effects, gravitational torque dependent on the joint angle, and external disturbances. The final joint angle is converted back to degrees for visualization, enabling accurate tracking of the desired knee motion through force-based actuation. To validate the control strategy and mechanical design prior to physical implementation, a comprehensive kinematic simulation was developed in MATLAB R2024a using Simscape Multibody [46] (Appendix A). The model comprises interconnected subsystems representing the mechanical structure and actuation mechanism. The world reference frame and coordinate transformation blocks define the initial configuration and gravity as a vector [0, 0, −9.8], while the leg structure is modeled through planar joints (Planar1, Planar2) and cylindrical joints (Cylindrical4, Cylindrical1) that replicate the anatomical degrees of freedom of the lower limb, including the thigh, shank, and foot segments. Revolute joints (Revolute2, Revolute3, Revolute4) represent the hip, knee, and ankle articulations. Quadriceps mechanism blocks (Quadriceps_calf_1, Quadriceps_calf_2, Quadriceps_support_1, Quadriceps_support_2) simulate the muscle-tendon actuation system, while the pneumatic actuation is represented by cylindrical rigid body blocks (Cylinder_1_RIGID, Fixed_Cap_1_RIGID) with force inputs controlled through signal blocks.

**Figure 7:**
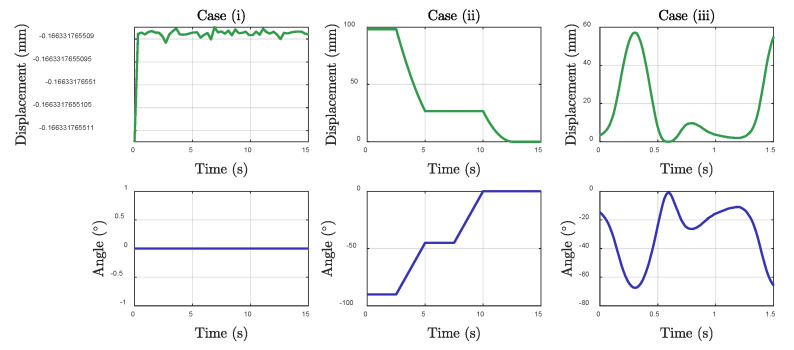
ExoKnee dynamic model following predefined trajectories with desired angles.

**Figure 8:**
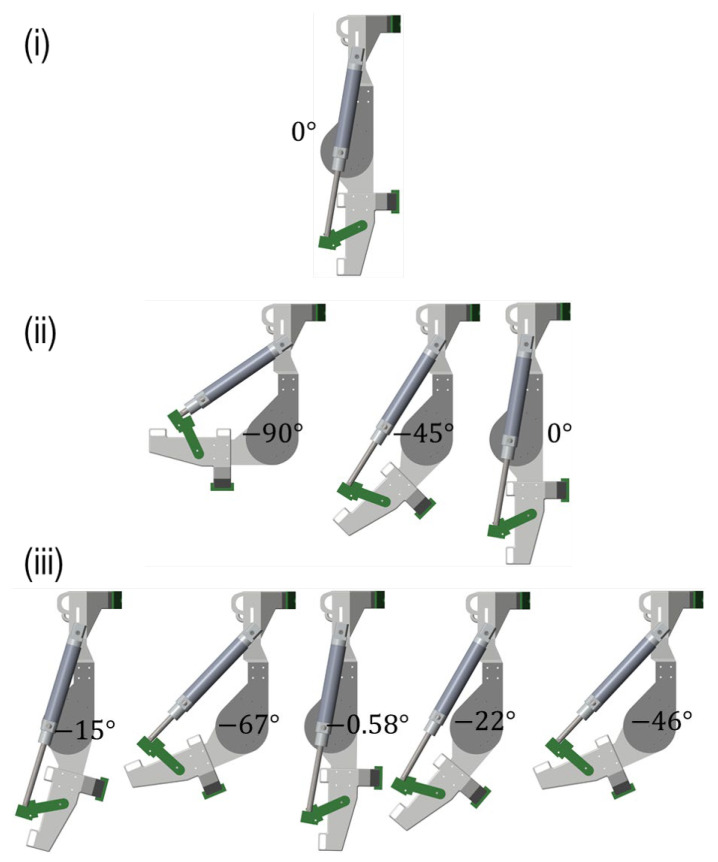
The ExoKnee prototype executes three validation scenarios: Case (**i**) maintains a constant −90° angle with stable steady-state behavior and negligible oscillations; Case (**ii**) implements stepwise transitions (−90° to −45° to 0°) at 2.5 s intervals, achieving smooth tracking and acceptable settling times; Case (**iii**) follows a sinusoidal trajectory representing physiological knee motion with bounded tracking error. These cases validate the control strategy’s robustness under static, piecewise-constant, and continuously varying references, confirming adaptability from static posture maintenance to dynamic gait assistance.

Three cases were considered for the mathematical model: (i) a constant knee angle reference of −90°; (ii) a stepwise reference signal changing from −90° to −45° and finally to 0°, with time intervals of 2.5 s between each transition; and (iii) a sinusoidal reference signal representing a physiological knee motion. For the first scenario, the system maintained a constant angular position of 0° with rod displacement oscillations below 10^−6^ mm, demonstrating effective steady-state regulation. For the second scenario, the system tracked each angular setpoint transition within the prescribed 2.5 s intervals, with rod displacement decreasing from 100 mm to 0 mm across successive steps and no observable overshoot. For the third scenario, the system followed a sinusoidal trajectory with angular displacement ranging between approximately −65° and −15° at a period of 0.6 s, with rod displacement varying between 0 and 58 mm. Overall, the three cases confirm the system’s ability to maintain static positions, track discrete transitions, and follow continuously varying references (Figure 7). ExoKnee executing the sequences of the three cases is shown in Figure 8.

**Figure 9:**
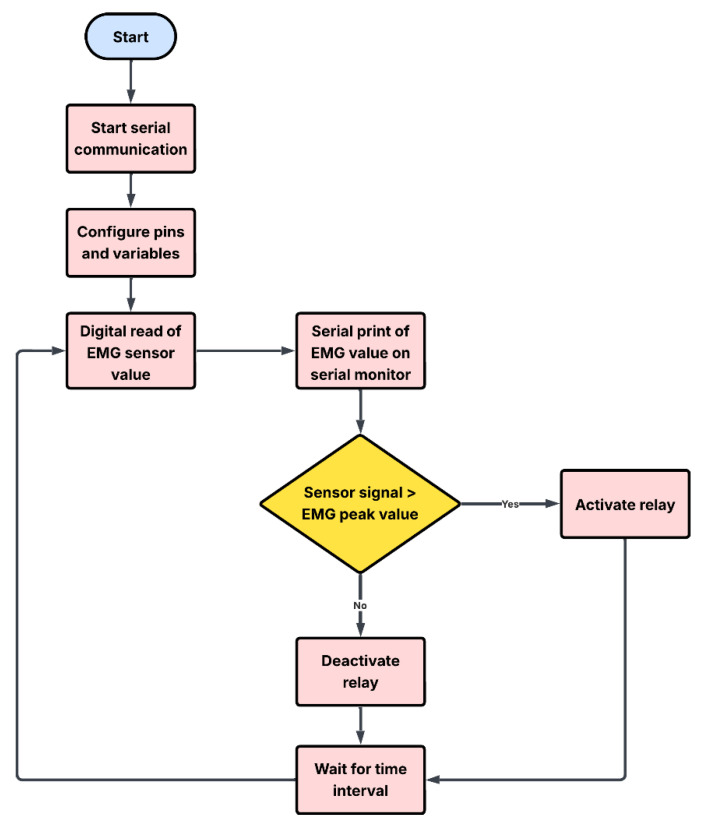
ExoKnee programming. The workflow initiates with serial communication setup and pin configuration, establishing hardware interfaces and defining critical parameters, including filter window size (15 samples) and EMG activation threshold (500). The system enters a continuous cycle beginning with digital acquisition of the EMG sensor value from the analog input, followed by signal processing through a moving average filter using a circular buffer to smooth raw electromyographic data. The filtered signal is transmitted via serial communication for real-time monitoring, then evaluated at a decision point, comparing the signal against the predefined threshold. If the sensor signal exceeds the EMG peak value, the system activates the relay to energize the exoskeleton actuator; otherwise, it deactivates the relay to maintain a passive state. The cycle concludes with a 100 ms delay to regulate the sampling rate before repeating, ensuring continuous EMG-based control of the ExoKnee device.

The Arduino IDE 2.3.4 software was selected for its enhanced stability and performance improvements over earlier versions. In the programming setup, analog pin A0 was assigned to capture the EMG signal, while pin 9 was configured as the relay communication port. To process the electromyographic signal readings, a variable was established to determine the maximum number of samples to be considered. Following this, global variables were declared to store the sensor reading array, the current index, the total sum of values, and the calculated average. The serial port configuration was set at a communication speed of 115,200 baud rate, with the EMG signal defined as input and the relay pin as output. The processing involved calculating and comparing the readings against the EMG signal peak value. The relay was triggered when the value exceeded the established average threshold and deactivated when it fell below this threshold, as illustrated in Figure 9 (Appendix B).

## 3. Results

### 3.1. Kinematics and Dynamics Analysis in a Therapy Routine

A standard therapy routine was considered for analysis. For the joint parameters, the joint position transitions from −90° to 0° over three intervals of 2.5 s each, then returns to −90° following the same time intervals. The joint velocity alternates between 0, 0.3 °/s, 0, and −0.3 °/s, indicating directional changes during the movement cycle. Joint acceleration exhibits peaks of ±125 °/s^2^ exclusively at velocity transitions.

The joint torque varies between approximately −3.5 N·m and +1.2 N·m throughout the therapy cycle, reflecting the gravitational loading during leg extension and the pneumatic assistive torque during knee flexion. These values are consistent with biomechanical estimates for assisted knee motion in elderly adults using the anthropometrically representative segment mass derived from Plagenhoef et al. [42]. The peak assistive torque of approximately 1.2 N·m is primarily constrained by the bore diameter of the selected pneumatic actuator (12 mm), which limits the available pneumatic force at 6-bar operating pressure. While this value is below the torque levels reported in active knee exoskeleton studies for full gait assistance, it is consistent with the proof-of-concept scope of this work. Future iterations should incorporate larger bore actuators or higher operating pressures to achieve clinically relevant assistive torque levels.

**Figure 10:**
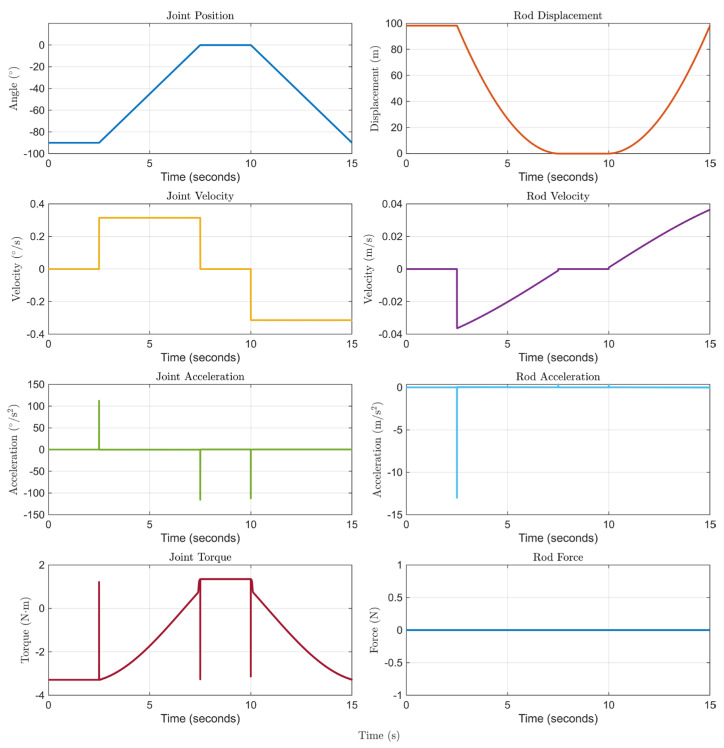
ExoKnee following a standard therapy routine.

For the rod parameters, the rod displacement decreases from its initial length of 100 mm to 0 mm, then returns to 100 mm. Rod velocity varies between −0.036 m/s and 0.036 m/s, corresponding to extension and retraction phases. Rod acceleration shows a single spike of −13.39 m/s^2^ at therapy initiation, while the rod force remains constant throughout the operation (Figure 10).

### 3.2. Expert Validation Assessment

To assess the feasibility and functionality of the proposed knee exoskeleton system, a validation process was conducted using expert judgment. Three engineers with expertise in mechanical, electrical, and mechatronic engineering were consulted to evaluate the system’s design, performance, and applicability. The expert panel consisted of two mechatronic engineers and one mechanical-electrical engineer, all of whom had extensive experience in developing biomechanical devices. A structured questionnaire was designed to systematically evaluate twelve key aspects of the exoskeleton, including mechanical design adequacy, anthropometric considerations, EMG-based control strategy, pneumatic actuation performance, safety criteria, kinematic modeling accuracy, and potential for clinical application in elderly users. Each item in the questionnaire was assessed according to four criteria: coherence (consistency with study objectives), clarity (absence of ambiguity), scale appropriateness (suitability of the evaluation metric), and relevance (importance to research goals). Experts rated each criterion on a five-point Likert scale ranging from 1 (unacceptable) to 5 (excellent). The primary objective of this validation was to determine whether the experts considered the system capable of being validated in real-world environments with elderly adults, based on its current design characteristics, functional performance, and safety features.

To quantitatively evaluate the expert responses, the Content Validity Coefficient (CVC) methodology was applied [47]. This statistical approach enables the measurement of consensus among a panel of experts (typically ranging from three to five) regarding both individual questionnaire items and the instrument as a whole. The calculation process begins by determining the mean score assigned by experts to each item, which is then divided by the maximum achievable score for that item, as shown in Equation (7).

(7) $$CVC_{i}=\frac{M_{x}}{V_{max}}$$

where Mx denotes the average rating provided by the expert panel for a particular item, and Vmax represents the highest possible score. To minimize potential bias introduced by individual evaluators, an error coefficient is computed for each item according to Equation (8).

(8) $${Pe}_{i}={\left(\frac{1}{j}\right)}^{j}$$

where j indicates the total number of experts involved in the validation process. The final Content Validity Coefficient is obtained by subtracting the error term from the initial coefficient, as expressed in Equation (9).

(9) $$CVC=CVC_{i}-Pe_{i}$$

Items achieving a CVC value exceeding 0.80 are deemed acceptable for retention in the validation instrument, though some researchers accept threshold values above 0.70. This quantitative framework provides an objective basis for determining whether each evaluated dimension of the exoskeleton system satisfies the necessary criteria for validation in practical clinical environments involving elderly populations.

The expert evaluation rubric comprised 12 criteria assessing: (1) structural adequacy of the mechanical design for knee flexion–extension; (2) appropriateness of materials and configuration for older adults; (3) anthropometric dimension considerations and joint alignment; (4) modular adaptability to different users; (5) suitability of EMG-based control for movement intention detection; (6) pneumatic actuation performance for smooth knee movements; (7) safety criteria for injury risk reduction; (8) biomechanically safe range of motion; (9) adequacy of kinematic and dynamic modeling; (10) validation through simulation and prototype testing; (11) potential as an assistive/rehabilitation device; and (12) capacity for future clinical validation and real-world application. Each criterion was evaluated across four dimensions: coherence, clarity, scale appropriateness, and relevance, using a 5-point Likert scale (Table 3).

## 4. Discussion

The content validity of the questionnaire was evaluated by three expert judges: Prof. J. A., Ph.D. (Appendix C), Prof. D. R., MSc. (Appendix C), and Prof. J. F., MSc. (Appendix C), all specialists in mechatronics and biomedical engineering. Each expert rated the 12 items on a scale from 1 to 5 based on four criteria: coherence, clarity, scale adequacy, and relevance. Table 3 presents the validity indices calculated for each item: Sx represents the sum of scores assigned by the three judges; Mx is the normalized score obtained by dividing Sx by the maximum possible score (20); CVCi (Content Validity Coefficient for each item) is calculated as Mx divided by the number of judges (3); Pei represents the probability of random error, calculated using Equation (8); and CVCtc (corrected Content Validity Coefficient) is obtained through Equation (9) to eliminate potential bias from chance agreement. According to the established interpretation criteria, the mean CVCtc value of 0.8747 indicates that the overall content validity of the instrument falls within the “good validity and concordance” range (0.80 < CVCtc < 0.90), indicating preliminary expert agreement on the conceptual design’s coherence, clarity, and relevance as evaluated through the structured instrument. It is important to note that this result reflects expert appraisal of the design concept and its theoretical foundations, not empirical measurement of device performance or therapeutic efficacy in real users. Three items fell below the 0.80 threshold: item 2 (materials and structural configuration, CVCtc = 0.7330), item 3 (anthropometric dimensions and joint alignment, CVCtc = 0.7163), and item 8 (biomechanically safe range of motion, CVCtc = 0.7497). These lower scores are consistent with the limitations acknowledged in this work regarding the need for expanded FEA validation, formal anthropometric sizing protocols, and clinical gait trials and will be prioritized in future development stages.

Among knee-specific exoskeletons reported in the literature, both Jiang et al. (2023) and Olinski (2025) address the kinematic complexity of the human knee by proposing mechanisms that track the instantaneous center of rotation (ICR) rather than simplifying the joint to a single-axis hinge. Jiang et al. implemented a five-bar mechanism with 2-DOF, achieving ICR trajectory errors below 5.52 × 10^−4^ m [25], while Olinski developed two polycentric prototypes, an adjustable crossed four-bar and a cam-based mechanism, capable of reproducing individual ICR trajectories with ROM variations not exceeding ±0.078 rad across repeated trials [48]. Despite their kinematic precision, both systems remain at a prototype stage without active actuation or functional gait assistance, and Jiang et al.’s system weighs approximately 14 kg, limiting viability for elderly users. In contrast, Zhou et al. (2023) and Chen et al. (2022) prioritized lightweight passive designs (1.16 kg and 567 g respectively), achieving knee torque reductions of 18.1% and modest EMG unloading (semitendinosus: 55.5% and 3.48%) [49,50], but their passive architectures lack the sustained assistive capacity required by older adults with significant quadriceps weakness.

Regarding actuation strategy, Aljarah et al. (2023) demonstrated that knee joint stiffness varies from approximately 30 Nm/rad during swing to 450 Nm/rad during early stance and that systems relying on fixed-stiffness actuators fail to track this profile across gait phases [51]. ExoKnee’s electro-pneumatic architecture addresses this by providing controllable assistive force adapted to EMG-detected muscular intent, prioritizing static holding capacity and structural robustness over dynamic bandwidth, a deliberate trade-off suited to gait stabilization in elderly adults. Furthermore, integrating an EMG-based reactive controller minimizes total system latency without the computational overhead of predictive models, offering a practical actuation pathway for community-level rehabilitation where neither high torque density nor GPU-level processing infrastructure is available.

At the clinical level, comparison with other systems oriented toward rehabilitation of older adults and post-stroke patients reveals relevant contributions. According to Gunnell et al., through a motorized knee exoskeleton that assists the affected joint via electromyographic control, an 8.8% reduction in sit-to-stand time was observed, along with a 13.7% increase in load symmetry and a 32% reduction in maximum activation of the quadriceps muscle on the affected side, as well as a 59% increase in total knee torque [52]. These results suggest a promising solution for improving standing ability, enhancing symmetry, reducing effort, and ultimately improving mobility and quality of life for stroke survivors. For their part, Lim et al. designed an ultralight hip exoskeleton with anti-phase torque, which showed a 14.8% and 10.6% increase in walking speed at 10 and 6 min respectively, in addition to approximately 75% increase in ankle dorsiflexion muscle strength. However, the absence of direct knee assistance restricts support during postural transition activities [53]. Similarly, Han et al. demonstrated the metabolic benefits following the use of a cable-driven soft wearable robot (SWR) for hip assistance, resulting in a 14.9% reduction in oxygen cost and a 0.11 m/s increase in speed during walking [54]. However, the knee joint was not subject to direct assistance, and the observed modifications in its kinematics correspond to secondary effects derived from the kinetic chain of gait.

Regarding robotic rehabilitation of the lower limbs, Fan et al. demonstrated that exoskeleton training in post-stroke patients induces significant changes in motor cortical excitability and neuroplasticity, evidenced by a reduction in resting motor threshold and increased motor evoked potentials, accompanied by functional improvements in gait and muscle coordination [6]. In a complementary manner, Guo et al., through a comprehensive review, highlighted that soft wearable exoskeletons optimize human–robot interaction and promote functional recovery by improving comfort, biomechanical adaptability, and control strategies based on movement intention [4]. Along the same lines, Feng et al. developed a portable isokinetic robot for knee rehabilitation, which showed substantial increases in muscle torque and work, consolidating the potential of wearable robotic systems as effective tools for progressive motor rehabilitation in clinical and subclinical settings [18]. The anthropometric design proposed by Martel Cervantes et al. demonstrated that precise measurements for correct biomechanical alignment of the knee and ankle are crucial for the development of functional and safe robotic systems, achieving knee flexion and extension movements within therapeutic ranges [3]. Likewise, the ergonomic considerations indicated by Christensen et al. in the development of the full-body exoskeleton FB-AXO show that misalignment between mechanical joints, as well as pressure exerted at anchor points, increases human–exoskeleton interaction forces and negatively affects the comfort and acceptability of the device in older adults [12]. In this context, ExoKnee, through its EMG-based control architecture, is designed to align actuation with the user’s motor intention. While this interaction has not yet been evaluated in gait trials, the proof-of-concept design provides a foundation for future investigation of naturalistic and efficient human–robot interaction.

It should be noted that the functional metrics reported above (including reductions in sit-to-stand time, improvements in gait speed, and metabolic cost reductions) were measured in the cited studies and have not been evaluated for ExoKnee in the present work. These comparisons are presented as a contextual reference to situate ExoKnee within the broader landscape of assistive knee devices, not to claim equivalent functional performance. Validation of ExoKnee against such outcome measures requires future clinical trials with elderly and post-stroke participants.

Finally, ExoKnee integrates mechatronic and biomedical features in a balanced manner as a more adaptable assistance system for the older adult population through its electromechanical design based on EMG control, allowing a synergistic response between muscular effort and robotic assistance, overcoming the limitations observed in purely passive devices or those with fixed control [21,24,52,53]. Likewise, its lightness, modularity, and ergonomic approach suggest potential viability for home-based and hospital rehabilitation programs, pending formal clinical evaluation, following contemporary principles of biomedical engineering and assistive robotics [3,4,6,55].

Compared with previously reported lower-limb assistive systems, ExoKnee presents a design profile oriented toward accessibility and local manufacturability. It is important to note that functional metrics such as gait speed improvement, metabolic cost reduction, or torque output have not yet been measured for ExoKnee; the comparisons presented here are therefore contextual, intended to situate the design within the existing literature rather than to assert equivalent performance. Clinical validation through gait trials with elderly users remains essential future work before any functional equivalence can be claimed.

From a technical perspective, ExoKnee’s pneumatic actuation strategy provides higher static force support compared with ultralight soft wearable systems, which typically generate forces around 19.1 N while weighing approximately 73 g. Although advanced technologies such as magnetorheological clutch systems have demonstrated torque densities up to 93.6 Nm/kg and machine learning-based intention detection models have achieved accuracies of 96.2%, these approaches introduce additional hardware complexity and computational delays of approximately 500–550 ms. By contrast, ExoKnee employs a simplified EMG-threshold controller that reduces computational latency while maintaining functional synchronization with muscle activation. However, pneumatic actuation introduces electromechanical delays near 220 ms, representing a trade-off between response speed and force capacity. These characteristics position ExoKnee as a balanced assistive solution emphasizing functional stability, accessibility, and feasibility for geriatric rehabilitation in resource-limited environments.

## 5. Conclusions

In conclusion, a proof-of-concept knee exoskeleton was designed, fabricated, and preliminarily evaluated, incorporating a pneumatic actuation system and EMG-based control using an AD8832 sensor to detect muscle activation signals that trigger a 24 V relay for knee flexion and extension. At this stage, the system demonstrated functional behavior in simulation and controlled mechanical response under single-subject threshold calibration, representing a promising foundation for future rehabilitation applications in elderly adults. The pneumatic system delivered consistent force output through the air compressor operating at 6-bar constant pressure, with flow regulation provided by choke valves to ensure smooth and controlled movements. The EMG threshold for activation was determined experimentally through a single-subject MVC calibration performed on one of the authors as a proof-of-concept procedure; future clinical application will require individualized calibration protocols.

With ethical approval already obtained from the Ethics Committee of the Institute of Research in Biomedical Sciences (INICIB) with code PI 013 2025 at Ricardo Palma University, and a patent application currently in progress, future work will focus on conducting clinical trials involving elderly patients and post-stroke survivors to validate therapeutic efficacy, usability, and long-term functional outcomes under real-world rehabilitation conditions. Additionally, future studies should expand biomechanical validation through finite element analysis incorporating dynamic loading scenarios, off-axis moments, cyclic fatigue analysis, and biomechanically representative loads derived from gait data, complementing the static structural evaluation already performed on the exoskeleton components. Improvements in control strategies are also required, including the implementation of normalized EMG calibration protocols based on individual maximum voluntary contraction percentage (%MVC), which are essential for accommodating inter-subject variability in muscle activation patterns, particularly in elderly users and post-stroke patients, where muscle weakness, spasticity, and signal variability are prevalent. Adaptive thresholding strategies and machine learning-based filtering represent promising directions for personalized calibration in future clinical trials.

Furthermore, additional characterization of the electro-pneumatic actuation system is necessary, including detailed evaluation of pneumatic cylinder response time, air pressure regulation precision, and operational acoustic noise, which represent critical parameters for clinical acceptance and patient comfort. Finally, future improvements should incorporate proportional pressure control using a pressure regulator with analog input, allowing EMG amplitude to modulate both extension velocity and final position. This approach would enable smoother, biomimetic movements that better accommodate the patient’s intention intensity while maintaining the safety boundaries established in this initial design.

## Figures and Tables

**Table 1:** Principal characteristics of knee exoskeletons.

Author	Characteristics	Advantages	Disadvantages
[31]	Robotic knee exoskeleton with event-based and adaptive control to improve mobility and rehabilitation.	Shows clinical feasibility in neuromotor impairment, improving gait stability and pattern while enabling repetitive, controlled neurorehabilitation training.	Limited by a small pilot sample, short-term evaluation, reduced adaptability for heterogeneous patients, and cost/accessibility constraints.
[32]	Features a bio-inspired variable stiffness actuator with parallel aramid fiber ropes and a seven-configuration clutch system, validated by viscoelastic and viscoplastic analysis.	Provides adaptive stiffness for improved comfort and compliance, with a lighter design and EMG evidence of reduced rectus femoris activation, indicating effective load sharing.	Limited by discrete stiffness modulation, mechanical clutch complexity, early rope stiffness softening, and a very small sample (n = 3).
[33]	Early post-stroke rehab device for bed-bound patients with a Series Elastic Actuator, precise encoder control, and preliminary mechanical and sEMG validation.	Offers compliant, intrinsically safe assistance via a Series Elastic Actuator, enabling early bed-bound rehabilitation with precise tracking and preliminary reduction in spasticity.	Limited by knee alignment dependency and 1-DoF hinge constraints that do not fully replicate natural joint motion, with only preliminary clinical validation in a small sample.
[34]	Knee exoskeleton assisting gait flexion through a human–robot model, combining active and passive elements for optimized energy storage and torque release.	Provides continuous gait assistance that enhances knee flexion, improves energy efficiency, optimizes human–robot coordination, and reduces muscular effort.	Limited by the need for individualized tuning, model-based assumptions, control complexity, and limited clinical validation.
[35]	Robotic gait exoskeleton with electric or hybrid actuators, integrated biomechanical sensors (angle, force, ±EMG), and adaptive or AI-based control for rehabilitation.	Improves mobility and gait, promotes neuroplasticity, and enables intensive rehabilitation while reducing therapist burden.	Limited by high cost, restricted accessibility, need for specialized training, device weight and bulk, battery dependence, and potential injury risk if improperly adjusted.
[36]	Active hip–knee–ankle exoskeleton with velocity-dependent torque modulation, optimized assistance profiles, and metabolic cost evaluation in healthy subjects.	Significantly reduces metabolic cost, dynamically adapts to different walking speeds, and enhances biomechanical efficiency during locomotion.	Complex multi-joint system with higher weight and energy consumption, validated only in healthy individuals and requiring speed-specific calibration.
[37]	Passive-elastic exoskeleton that stores energy during knee extension and releases it to assist ankle plantarflexion, with evaluation of walking metabolic cost.	Lightweight, energy-efficient design without active actuators, reducing metabolic demand, electronic complexity, and overall power consumption.	Limited by non-adaptive assistance without active control, reliance on passive mechanical synchronization, and minimal real-time personalization.
[38]	Unilateral active knee exoskeleton with programmable assistive/resistive modes, evaluated in post-stroke patients using flexion range and EMG metrics.	Improves knee flexion range and neuromuscular activation, showing potential for motor rehabilitation applications.	Limited by its unilateral design without bilateral assessment, small sample size, and the need for a complex active control system.
[39]	Anthropometrically parameterized design optimizing joint alignment and torque transmission, with strong structural and mechanical emphasis.	Improves joint alignment precision, reducing misalignment and discomfort while enhancing mechanical efficiency in force transmission.	Limited by the lack of advanced control strategies, design-focused validation with minimal clinical outcome data, and the potential need for complex individual customization.
[40]	Hip–knee coupled exoskeleton based on offset theory, enabling coordinated energy transfer and phase-dependent gait assistance.	Enhances inter-joint interaction, reduces mechanical discontinuities during walking, and provides biomechanically coordinated assistance.	Mechanically complex system that relies on precise gait-phase synchronization and has been primarily validated in experimental settings.

**Table 2:** Physical and Geometric Parameters of the ExoKnee Prototype.

Parameter	Symbol	Value	Unit
Thigh anchoring length	$r_{thigh}$	0.173	m
Shank anchoring length	$r_{leg}$	0.180	m
Structural offset angle	α	3.71	Degrees
Mass of the moving segment	$M_{sys}$	3.85	kg
Distance to the center of mass	$d_{cm}$	0.077	m
Equivalent inertia	$I_{eq}$	0.4	kg·m^2^
Viscous friction coefficient	B	Variable	N·m·s/rad
Piston diameter	$D_{p}$	9.90	mm
Actuator stroke	S	100	mm
Nominal operating pressure	$P_{nom}$	6.00	bar

**Table 3:** Expert validation questionnaire results.

Expert	Expert 1	Expert 2	Expert 3	Sx	Mx	CVCi	Pei	CVCtc
Item								
1	16	20	18	54	2.7	0.9000	0.0003	0.8997
2	15	11	18	44	2.2	0.7333	0.0003	0.7330
3	16	11	16	43	2.15	0.7167	0.0003	0.7163
4	17	20	20	57	2.85	0.9500	0.0003	0.9497
5	18	20	20	58	2.9	0.9667	0.0003	0.9663
6	17	20	20	57	2.85	0.9500	0.0003	0.9497
7	16	20	16	52	2.6	0.8667	0.0003	0.8663
8	18	11	16	45	2.25	0.7500	0.0003	0.7497
9	16	20	17	53	2.65	0.8833	0.0003	0.8830
10	18	20	18	56	2.8	0.9333	0.0003	0.9330
11	16	20	20	56	2.8	0.9333	0.0003	0.9330
12	19	20	16	55	2.75	0.9167	0.0003	0.9163
							Mean	0.8747

## Data Availability

The original contributions presented in the study are included in the article. Further inquiries can be directed to the corresponding author.

## Appendix B

### ExoKnee Programming

**Parameter**	**Symbol**	**Value**
Parameter definition	const int emgPin = A0; const int relayPin = 8; const int filterWindow = 15; const int emgThreshold = 500;	Definition of the analog input used for EMG acquisition, the digital output for relay control, the size of the moving average window, and the activation threshold.
Signal buffer declaration	int emgSamples[filterWindow]; int sampleIndex = 0; int sampleSum = 0; int emgFiltered = 0;	Declaration of variables required for implementing a moving average filter using a circular buffer approach.
Hardware initialization	Serial.begin(115200); pinMode(emgPin,INPUT); pinMode(relayPin, OUTPUT);	Initialization of serial communication for monitoring and configuration of microcontroller pins for sensor input and actuator output.
Buffer initialization	for (int i = 0; i < filterWindow; i++) { emgSamples[i] = 0; }	Initialization of the signal buffer to zero in order to avoid transient artifacts during system startup.
Removal of oldest sample	sampleSum -= emgSamples[sampleIndex];	The oldest EMG sample is removed from the accumulated sum to maintain a constant window size.
EMG acquisition	emgSamples[sampleIndex] = analogRead(emgPin);	Acquisition of a new EMG sample using the analog-to-digital converter of the microcontroller.
Buffer update	sampleSum += emgSamples[sampleIndex]; sampleIndex++; if (sampleIndex >= filterWindow) { sampleIndex = 0; }	Update of the circular buffer index and inclusion of the new EMG sample in the accumulated sum.
Signal smoothing	emgFiltered = sampleSum/filterWindow;	Computation of the moving average to obtain a smoothed representation of the EMG signal.
Signal monitoring	Serial.print(“Filtered EMG:”); Serial.println(emgFiltered);	Transmission of the filtered EMG value through serial communication for real-time monitoring and debugging.
Decision logic	if (emgFiltered >= emgThreshold){	Comparison of the filtered EMG signal with a predefined threshold representing sufficient muscle activation.
Relay activation	digitalWrite(relayPin, HIGH);	Activation of the relay when the EMG activation threshold is exceeded.
Relay deactivation	digitalWrite(relayPin, LOW);	Deactivation of the relay when the EMG signal remains below the activation threshold.
Sampling control	delay(100); }	Introduction of a fixed delay to regulate the sampling rate and ensure stable system operation.
